# Supplementary material for: Multi‐dataset identification of innovative feature genes and molecular mechanisms in keratoconus
Source: J Cell Mol Med. 2024 Sep 19;28(18):e70079. doi: 10.1111/jcmm.70079 (PMC11412914; doi:10.1111/jcmm.70079)
Supplement: Supplementary file 2 — Material S2. [file JCMM-28-e70079-s002.zip › 7.GSEA analysis/6.SNORA74B/KEGG_THYROID_CANCER.html]

Details for gene set KEGG\_THYROID\_CANCER[GSEA]

|  || Dataset | SNORA74B.group.cls#h\_versus\_l |
| Phenotype | group.cls#h\_versus\_l |
| Upregulated in class | l |
| GeneSet | KEGG\_THYROID\_CANCER |
| Enrichment Score (ES) | -0.43841687 |
| Normalized Enrichment Score (NES) | -1.5762873 |
| Nominal p-value | 0.028248588 |
| FDR q-value | 0.30675265 |
| FWER p-Value | 0.651 |
Table: GSEA Results Summary

  

Fig 1: Enrichment plot: KEGG\_THYROID\_CANCER      
 Profile of the Running ES Score & Positions of GeneSet Members on the Rank Ordered List

  

| SYMBOL | TITLE | RANK IN GENE LIST | RANK METRIC SCORE | RUNNING ES | CORE ENRICHMENT || 1 | RET | na | 3274 | 0.288 | -0.0135 | No |
| 2 | BRAF | na | 8089 | 0.134 | -0.1147 | No |
| 3 | KRAS | na | 9766 | 0.097 | -0.1357 | No |
| 4 | NTRK1 | na | 10524 | 0.083 | -0.1342 | No |
| 5 | RXRA | na | 12303 | 0.056 | -0.1698 | No |
| 6 | TCF7L2 | na | 12663 | 0.051 | -0.1658 | No |
| 7 | MAP2K2 | na | 14904 | 0.025 | -0.2232 | No |
| 8 | CDH1 | na | 15137 | 0.023 | -0.2234 | No |
| 9 | CCND1 | na | 15223 | 0.022 | -0.2196 | No |
| 10 | NCOA4 | na | 15745 | 0.017 | -0.2298 | No |
| 11 | MAPK3 | na | 16753 | 0.007 | -0.2569 | No |
| 12 | CCDC6 | na | 17442 | 0.000 | -0.2767 | No |
| 13 | CTNNB1 | na | 17783 | -0.003 | -0.2855 | No |
| 14 | TPM3 | na | 18657 | -0.013 | -0.3069 | No |
| 15 | TP53 | na | 19572 | -0.024 | -0.3265 | No |
| 16 | HRAS | na | 19699 | -0.025 | -0.3231 | No |
| 17 | MAPK1 | na | 20606 | -0.035 | -0.3393 | No |
| 18 | TFG | na | 22067 | -0.053 | -0.3666 | No |
| 19 | PAX8 | na | 23291 | -0.070 | -0.3823 | No |
| 20 | RXRB | na | 24595 | -0.091 | -0.3942 | No |
| 21 | MYC | na | 25534 | -0.108 | -0.3909 | No |
| 22 | TCF7L1 | na | 27185 | -0.144 | -0.3981 | Yes |
| 23 | TPR | na | 27863 | -0.161 | -0.3723 | Yes |
| 24 | NRAS | na | 27969 | -0.164 | -0.3293 | Yes |
| 25 | TCF7 | na | 28810 | -0.189 | -0.3003 | Yes |
| 26 | MAP2K1 | na | 31129 | -0.275 | -0.2899 | Yes |
| 27 | PPARG | na | 31596 | -0.300 | -0.2190 | Yes |
| 28 | RXRG | na | 33623 | -0.467 | -0.1461 | Yes |
| 29 | LEF1 | na | 34351 | -0.633 | 0.0109 | Yes |
Table: GSEA details [plain text format]

  

Fig 2: KEGG\_THYROID\_CANCER      
 Blue-Pink O' Gram in the Space of the Analyzed GeneSet

  

Fig 3: KEGG\_THYROID\_CANCER: Random ES distribution      
 Gene set null distribution of ES for **KEGG\_THYROID\_CANCER**
